# Assessment of Behavioral Health Readiness for Gender-Affirming Surgery: A Cross-Sectional Survey of Mental Health Provider Practices

**DOI:** 10.7759/cureus.107604

**Published:** 2026-04-23

**Authors:** Madyson Brown, Ferris Zeitouni, Essie Ghafoor, Bashar Hassan, Calvin R Schuster, Mona Ascha, Romy Smith, Kate Thomas, Zackary Berger, Fan Liang

**Affiliations:** 1 Department of Plastic and Reconstructive Surgery, Center for Transgender and Gender Expansive Health, Johns Hopkins University School of Medicine, Baltimore, USA; 2 Division of Plastic and Reconstructive Surgery, University of Illinois College of Medicine at Chicago, Chicago, USA; 3 Department of General Internal Medicine/Johns Hopkins Berman Institute of Bioethics, Johns Hopkins University School of Medicine, Baltimore, USA

**Keywords:** gender-affirming surgery, mental health, surgical readiness assessment, transgender and nonbinary, wpath standards of care

## Abstract

Introduction

Gender-affirming surgery (GAS) is associated with improved mental health and quality of life outcomes for transgender and nonbinary (TGNB) individuals; however, access to GAS often requires preoperative behavioral health readiness evaluations, as outlined by the World Professional Association for Transgender Health, which result in a letter of surgical readiness. Little is known about how mental health providers (MHPs) conduct preoperative readiness assessments in current practice. This cross-sectional study assessed how MHPs evaluate surgical readiness prior to GAS and examined differences in assessment approaches by provider training, experience, and gender identity.

Methods

A 25-question survey was developed by the research team and administered electronically using the Johns Hopkins University Research Electronic Data Capture (REDCap) platform. Eligible participants included MHPs in Maryland and the District of Columbia listed on Psychology Today who identified "transgender" as an area of competency. Responses were grouped by respondent training, evaluation volume, and gender identity. Assessment methods, prioritized psychosocial domains, and potential referral barriers were compared using Chi-square/Fisher’s exact tests. The number of sessions and assessment outcomes between groups were compared with one-way analysis of variance. The survey was distributed to 462 unique email addresses and remained open for six weeks from October 14 to November 30, 2022.

Results

Of 462 MHPs, the response rate was 21% (n=97). Among respondents with complete data for both variables, MHPs who attended conferences to augment their training found more value in the letter-writing process compared to those who did not attend conferences (n=29/46 (63%) vs. *n*=9/28 (32.1%); p=0.028). The inability to provide informed consent (60.8%, n=59/97) and unrealistic expectations (46.4%, *n*=45/97) were the most frequently cited factors that would preclude a letter of surgical readiness. A significantly higher proportion of TGNB MHPs considered unrealistic expectations to be a contraindication to referral compared to cisgender MHPs (n*=*16/18 (88.9%) vs. *n*=29/56 (51.8%); p=0.005). The median (interquartile range) number of sessions needed between letter request and referral for surgery was 2 (1-4). A median (interquartile range) of 95% (85%-100%) of clients were granted referral.

Conclusion

Approaches to preoperative evaluation varied. Experienced, high-volume MHPs and TGNB MHPs emphasized the capacity for consent, autonomy, and realistic surgical expectations over their peers. Formalized training in TGNB care increased perception of letter value.

## Introduction

Gender-affirming surgery (GAS) can help transgender and nonbinary (TGNB) individuals align their physical appearance with their gender identity. Access to GAS has been shown to improve psychological well-being and quality of life [1-3]. Despite growing demand, access remains limited [4,5]. The World Professional Association for Transgender Health (WPATH) Standards of Care, version 7 (SOCv7), recommended one or more behavioral health (BH) letters, often from a doctoral-level provider, before surgery [6]. These requirements were widely adopted by insurance companies and have persisted despite updates in SOCv8 advocating for one referral letter from any qualified, master’s-level professional [7].

In practice, the BH readiness assessment is a preoperative clinical evaluation that typically includes confirmation of gender incongruence, review of any mental health conditions and their management, assessment of capacity for informed consent, discussion of fertility implications, and evaluation of psychosocial support and practical readiness for postoperative recovery [7,8]. These evaluations typically result in a BH readiness letter that summarizes these domains and communicates to the surgical team that the patient has undergone a comprehensive assessment within a multidisciplinary framework [8]. Critics of the BH letter argue that the process promotes gatekeeping, places insufficient emphasis on informed consent, and evaluator bias [9-12]. With the SOCv8 updates, the letter intent was reframed as a collaborative assessment to establish a diagnosis, surgical eligibility, and overall readiness [7].

Patients pursuing bariatric surgery are similarly required to undergo a formal psychosocial-behavioral evaluation by a mental health provider (MHP). In the absence of standardized guidelines, studies have identified wide variability in assessment practices [13]. Although the literature is limited on considerations for MHPs working with TGNB patients, the lack of training remains a concern [14-18]. In particular, the evaluation strategies used by MHPs when writing readiness letters have not been well studied [14,17,18].

Given the diverse training backgrounds of MHPs and the variability in letter-writing practices, we aimed to characterize the strategies and psychosocial domains evaluated by MHPs during presurgical assessments for GAS referral. We distributed a survey to providers in the Baltimore, Maryland, and Washington, D.C. areas. We hypothesized that providers with greater letter-writing experience and those who identify as TGNB would demonstrate a more structured approach. This study seeks to promote more consistent, effective, and sensitive evaluation practices for TGNB individuals pursuing GAS.

## Materials and methods

We developed an anonymous, online, cross-sectional survey for MHPs who evaluate TGNB individuals for GAS referral. The inclusion criteria were providers listed on Psychology Today who identified “transgender” as an area of competency and were located in Maryland or Washington, D.C. These regions were chosen for their relatively large TGNB populations, 0.92% in Washington, D.C. and 0.51% in Maryland [19]. The exclusion criteria were providers with missing, duplicate, or invalid emails. Providers without a relevant advanced degree (Doctor of Philosophy (PhD), Doctor of Psychology (PsyD), Doctor of Education (EdD), Master of Social Work (MSW), Licensed Clinical Social Worker (LCSW), Master of Arts (MA), Master of Science (MS), Doctor of Medicine (MD), Doctor of Osteopathic Medicine (DO), Registered Nurse (RN), Master of Science in Nursing (MSN), Certified Registered Nurse Practitioner (CRNP)) were also excluded from our analysis. Ethical approval was obtained from the Johns Hopkins School of Medicine Electronic Institutional Review Board (eIRB2; approval IRB00347595).

The primary objective of this study was to characterize current BH readiness assessment practices used by MHPs during presurgical evaluations for GAS, including assessment strategies, prioritized psychosocial domains, and referral-related practices. Secondary objectives were to examine whether these practices differed according to provider training, evaluation volume, and gender identity. The survey consisted of 25 items and was estimated to take 15 minutes to complete. It included questions about demographics, professional training, practice characteristics, prior experience, surgical readiness assessment content, readiness assessment practices, and outcomes of assessments. Demographic and practice variables were adapted from similar surveys in transplant and bariatric surgery literature [13,18,20]. The included domains were chosen based on WPATH SOCv7 and SOCv8 guidelines regarding recommended letter content [6,7].

The demographics section of the survey asked about respondent gender, race, ethnicity, and age. The professional training section included professional title, highest level of education, and prior training in caring for transgender patients. Practice characteristics included familiarity with and use of WPATH SOC, practice type (public or private), and practice setting (urban, suburban, rural, or telehealth). Prior experience included years in practice, years spent conducting evaluations, and the average number of surgical readiness assessments conducted per year for gender-affirming surgery [6,7].

In this study, surgical readiness assessment refers to the presurgical mental health evaluation conducted to inform referral for gender-affirming surgery. The survey section on surgical readiness assessment content inquired about procedures utilized and frequency of use, domains assessed and frequency of inclusion, the three most important domains assessed, and discussion of postoperative depression during the assessment. The section on readiness assessment practices included hours spent with the patient during the evaluation, the number of sessions before writing a letter of surgical readiness, and perceived value in the current referral process. Finally, the section on outcomes of assessments included referral contraindications, permanent barriers to surgical referral, inclusion of explicit recommendations in the letter, and the proportion of assessments resulting in referral. A copy of the survey and recruitment messaging is available in the Appendix.

Survey data were collected and managed using the Johns Hopkins University Research Electronic Data Capture (REDCap) database. Of 839 providers identified on Psychology Today, 377 were excluded due to missing, duplicate, or invalid email addresses. The survey was distributed to 462 email addresses and remained open for six weeks from October 14, 2022, to November 30, 2022. A total of four reminder emails were sent to nonrespondents on days 7, 14, 29, and 42. Participants who completed the survey were eligible to enter a raffle for a $200 gift card. This study was designed as an exploratory, cross-sectional survey among MHPs conducting surgical readiness evaluations for GAS. Therefore, no formal a priori sample size or power calculation was performed. Instead, the sampling strategy aimed to survey the full, accessible population of eligible providers practicing in Maryland and Washington, D.C., as identified through a public professional directory. The final sample size was therefore determined by the size of the sampling frame and the observed response rate.

MHPs were grouped by method of training in TGNB care, number of evaluations completed per year, and gender identity. High-volume providers were defined as those in the top 25th percentile for annual number of letters written, corresponding to 10 or more letters per year. Respondent gender was categorized as cisgender or TGNB. Variables included in the subgroup analysis were as follows: most important domains assessed, discussion of post-operative depression, issues to address before referral, perceived value of the assessment process, permanent barriers, number of sessions required before referral, and assessment outcomes.

Normally distributed data were reported as mean ± standard deviation (SD), and non-normally distributed data as median with interquartile range (IQR). Bivariate analyses were performed to compare the number of sessions required, the psychosocial domains emphasized, and assessment outcomes between TGNB versus cisgender MHPs and high-volume (top 25th percentile) versus low-volume MHPs. Continuous variables were analyzed using one-way analysis of variance, and categorical variables using Chi-square or Fisher’s exact tests. Analyses were performed using SPSS software, version 29 (IBM Corp., Armonk, NY), with p < 0.05 considered statistically significant [21].

## Results

Respondent characteristics

Our survey yielded 98 responses; one was excluded for lack of an advanced degree, leaving 97 respondents included in our analysis. Table 1 summarizes respondent demographics and practice characteristics. The median (IQR) age was 38 (34-44) years. Most were cisgender women (n=54/97, 55.7%), followed by cisgender men (n=21/97, 21.6%), nonbinary individuals (n=20/97, 20.6%), and transgender men (n=2/97, 2.1%). None identified as transgender women. The majority were non-Hispanic White (n=73/97, 75.2%) and practiced in private settings (n=81/96, 84.4%). Respondents were primarily social workers (n=37/97, 38.1%) and psychologists (n=31/97, 32.0%).

**Table 1:** Respondent characteristics IQR: interquartile range. SD: standard deviation. TGNB: transgender or nonbinary. NHOPI: Native Hawaiian and Other Pacific Islander people. MSW: Master of Social Work. LCSW: Licensed Clinical Social Worker. PhD: Doctor of Philosophy. PsyD: Doctor of Psychology. EdD: Doctor of Education. MA: Master of Arts. MS: Master of Science. RN: Registered Nurse. MSN: Master of Science in Nursing. CRNP: Certified Registered Nurse Practitioner. WPATH SOC: World Professional Association Standards of Care. MD: Doctor of Medicine. DO: Doctor of Osteopathic Medicine. LCMFT: Licensed Clinical Marriage and Family Therapist.

Respondent Characteristics					
Total (n=97)					
	Median	IQR		Mean	SD
Age in years	38	34-44	Percentage of evaluations that result in:		
Average conducted per year	2	1-5	Approval for surgery	88	20
Percentage of assessments resulting in a surgical referral.	95	85-100	Deferral until specific criteria are met	9	15
Years in Practice	7	4-11	Foregoing surgery	1	3
Years of conducting assessments	5	2-7			
How many sessions do you require before you provide a letter?	2	1-4			
Characteristics	N	%	Characteristics	N	%
Gender (N=97)			Training in TGNB care (N=97)		
Cisgender Woman	54	55.7	Continuing education	81	83.5
Cisgender Man	21	21.6	Professional conferences	57	58.8
Transgender Man	2	2.1	Training during residency, fellowship, internship, or other clinical setting	46	47.4
Transgender Woman	0	0.0	Mentorship	42	43.3
Non-Binary	20	20.6	Graduate course	29	29.9
Race and Ethnicity (N=97)			Other	17	17.5
White	73	75.2	Consultation	6	6.2
Black	15	15.5	Literature	4	4.1
Hispanic or Latinx	11	11.3	Certification	4	4.1
Asian	7	7.2	Lived experience	2	2.1
NHOPI	1	1.0	Educated by clients or the community at large	7	7.2
More than 1 race	10	10.3	Assessment Strategies and Outcomes (N=74)		
Professional Category (N=97)			Permanent Barriers Exist		
Social Worker	37	38.1	Yes	29	39.2
Psychologist	31	32.0	No	27	36.5
Counselor	25	25.8	Unsure	18	24.3
Psychiatric Nurse	3	3.1	Do you perceive value in the current referral process? (N=74)		
Psychiatrist	1	1.0	Yes	38	51.4
Practice Setting (N=96)			No	10	13.5
Private	81	84.4	Unsure	26	35.1
Public	15	15.6	Highest Level of Education (N=97)		
Practice Type (N=96)			MSW, LSCW	37	38.1
Telehealth	41	42.7	PhD, PsyD, EdD	36	37.1
Urban	39	40.6	MA, MS	20	20.6
Suburban	14	14.5	RN, MSN, CRNP	3	3.1
Rural	2	2.1	MD, DO	1	1.0
Familiar with WPATH SOC	74	76.2	LCMFT	0	0.0
Use WPATH SOC	68/74	91.9			

**Figure 1:**
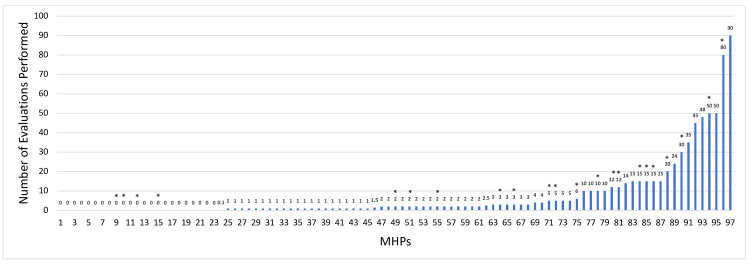
Distribution of evaluations performed per year Histogram of evaluations per provider per year. The median (interquartile range) number of evaluations is 2 (1-5). The 75th percentile of evaluations is 10. Greater than 70% of letter writers provided fewer than five letters per year. Asterisks indicate mental health providers who identified as transgender or nonbinary.

Respondents had a median (IQR) of 7 (4-11) years in practice and 5 (2-7) years of experience conducting surgical readiness assessments. Median (IQR) annual number of assessments was 2 (1-5), ranging from 0 to 90 (Figure 1). Over 70% of respondents completed fewer than five assessments per year. Twenty-two MHPs conducted 10 or more assessments annually and were classified as high-volume providers (in the top 25th percentile). Among respondents familiar with WPATH SOC (n=74/97, 76.2%), most (n=68/74, 91.9%) reported using the SOC in their practice.

Prior training

Among respondents, the most common type of TGNB-focused training was participation in continuing education (n=81/97, 83.5%), followed by professional conferences (n=57/97, 58.8%), training in a clinical setting (n=46/97, 47.4%), and graduate courses (n=29/97, 29.9%) (Table 1). In the free-response section, the most frequent answer was learning from clients or working in the community (n=7/17, 41.2%) (Table 1). Additional responses included lived experience, WPATH membership, self-study of current literature and SOC guidelines, and supervision or consultation with experienced providers. Most respondents held MS/LCSW credentials (n=37/97, 38.1%), followed by PhD/PsyD/EdD (n=36/97, 37.1%), MA/MS (n=20/97, 20.6%), RN/MSN/CRNP (n=3/97, 3.1%), and MD/DO (n=1/97, 1%) (Table 1).

Evaluation practices

**Figure 2:**
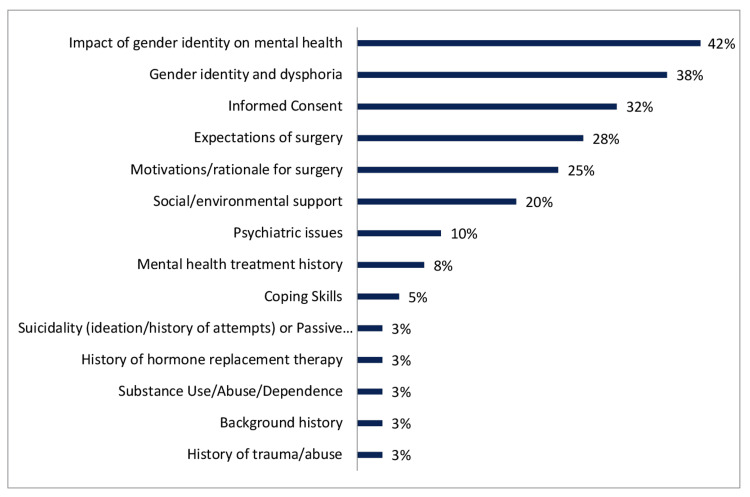
Responses to most important domains assessed Providers were asked to choose from 17 evaluation domains to signify the top three domains they found to be most important for assessment during surgical readiness evaluation.

Survey respondents were allowed to choose from 17 domains to identify the three most important evaluation considerations during presurgical assessments. Several noted that limiting the selection to three was challenging, as all domains were frequently addressed. Nearly half identified the impact of gender identity on mental health as the most important (n=41/97, 42%), followed by understanding the patient’s gender identity and dysphoria (n=37/97, 38%) and capacity for informed consent (n=31/97, 32%) (Figure 2).

**Figure 3:**
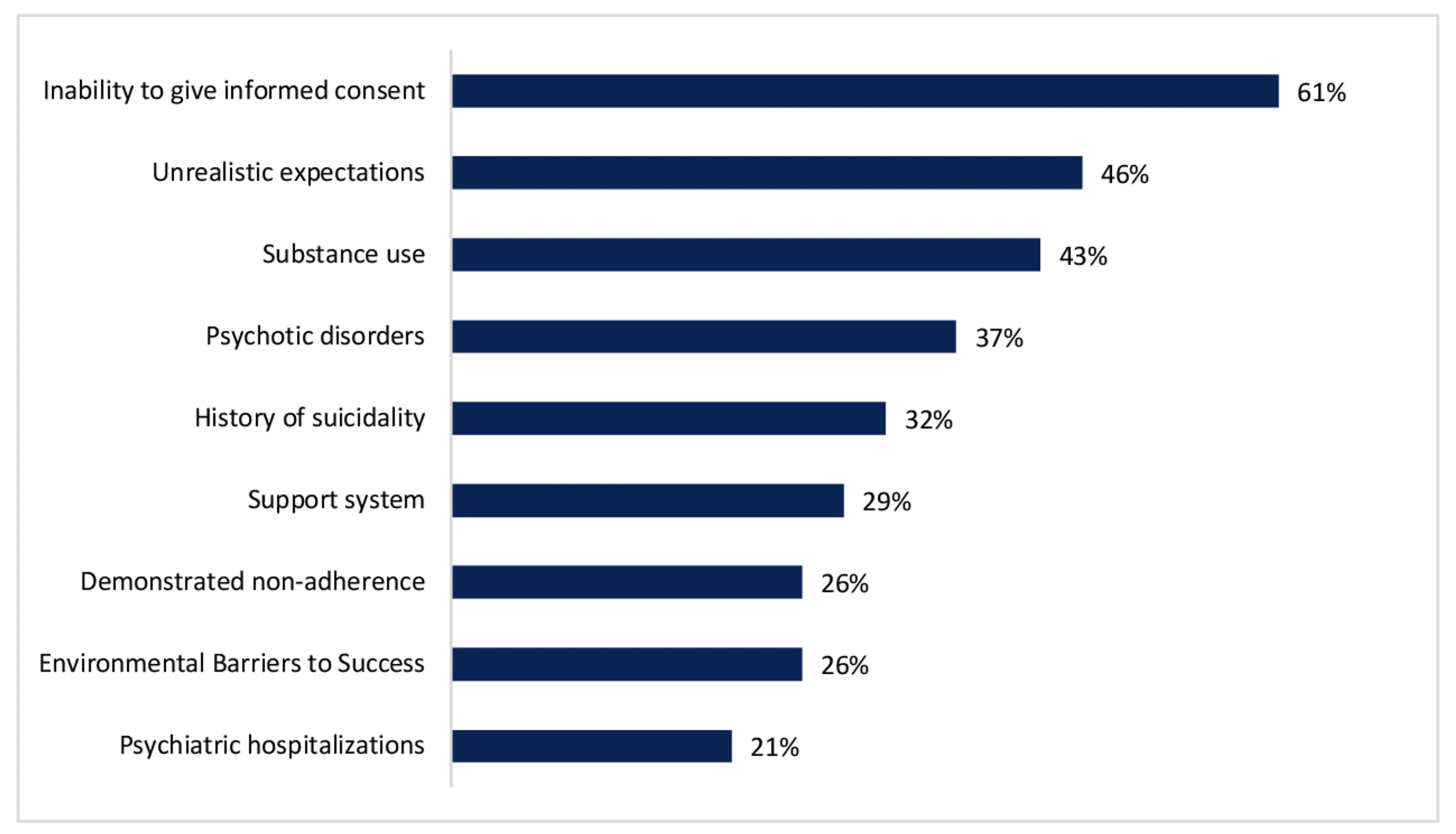
Issues to address before surgery This figure demonstrates the most common issues that required optimization prior to surgery, as reported by mental health providers.

Other commonly emphasized domains included patient expectations for surgery (n=27/97, 28%), motivations for surgery (n=24/97, 25%), and social/environmental support (n=20/97, 20%). Write-in responses highlighted post-surgical recovery planning, autonomy, treatment adherence barriers, financial constraints, expectations for life change following surgery, history of trauma, gender journey, and social transition steps, and understanding the impact of surgery on sexual function. The most frequent issues requiring optimization prior to surgery were inability to give informed consent (n=59/97, 60.8%), unrealistic expectations (n=45/97, 46.4%), and substance use (n=42/97, 43.3%) (Figure 3).

High-volume practices were more common among TGNB MHPs compared to cisgender MHPs (n=10/22, 45.5% vs. n=12/75, 16.0%; p=0.004). Compared to low-volume MHPs, high-volume MHPs were more likely to have attended a professional conference (n=18/22, 81.8% vs. n=39/75, 52.0%; p=0.012) and received mentorship (n=17/22, 77.3% vs. n=25/75, 33.3%; p<0.001). No significant association was found between TGNB identity and specific training experiences.

Assessment outcomes

In total, 29 (29.9%) MHPs reported a belief that there are issues that are permanent barriers to referral for GAS, 27 (27.8%) did not, and 18 (18.6%) were unsure. Evaluations resulted in surgical referral for a median (IQR) of 95% (85-100%) of clients (Table 1), with neither MHP volume nor gender identity significantly affecting referral rates. On average, 88% of evaluations led to referral, 9% to conditional referral if specific issues were addressed, and 1% to non-referral. The mean (SD) and median (IQR) number of sessions before referral were 3.13 (2.87) and 2 (1-4), respectively. Seven (7.2%) MHPs only provided letters to established clients. Of the 74 respondents to this question, most MHPs (n=54/74, 73%) reported that they made explicit recommendations of endorsement or non-endorsement for patients to undergo GAS in their referral letters. Of 74 MHPs reporting on the perceived value of behavioral health assessments, 38 (51.4%) saw value, 26 (35.1%) were unsure, and 10 (13.5%) saw no value.

Subgroup comparisons by provider gender and practice volume

Subgroup analysis was performed to examine how MHP training, experience, and gender identity influenced assessment approaches. Table 2 shows domains emphasized stratified by MHP training type. Of those who completed the section on key domains assessed, MHPs who underwent continuing education placed greater emphasis on establishing clear surgical expectations (n=27/66 vs. n=0/8; p=0.024), while those with mentorship placed less emphasis on patients’ mental health history (n=1/39 vs. n=7/35; p=0.023) in establishing surgical readiness.

**Table 2:** Most important domains stratified by training experience This table displays the proportion of mental health providers (MHPs) who identified specific psychosocial domains as among the three most important components of preoperative behavioral health assessments for gender-affirming surgery, stratified by type of training (attendance at professional conferences, receipt of mentorship, participation in continuing education, clinical training, and completion of graduate coursework). The table also reports perceived value of the readiness letter process (yes, no, unsure), practice volume classification (low volume or high volume), and gender identity of MHP (cisgender or transgender) within each training category. Mean (± standard deviation) number of sessions required prior to letter writing and mean (± standard deviation) proportion of assessments resulting in surgical referral are shown for each training category. Frequency data are reported as No. (%), with percentages calculated using respondents with available data for each item; therefore, denominators may vary by row because missing responses were excluded from row-specific analyses.

Domain Assessed	Training Experience														
	Professional Conference			Mentorship			Continuing Education			Clinical Training			Graduate Course		
	No n (%)	Yes N=46 n (%)	p	No n (%)	Yes N=39 n (%)	p	No n (%)	Yes N=66 n (%)	p	No n (%)	Yes N=35 n (%)	p	No n (%)	Yes N=23 n (%)	p
Background history	2 (7.1)	1 (2.2)	0.553	2 (5.7)	1 (2.6)	0.600	1 (12.5)	2 (3.0)	0.294	1 (2.6)	2 (5.7)	0.600	2 (3.9)	1 (4.3)	1.0
Trauma history	1 (3.6)	2 (4.3)	1.0	3 (8.6)	0 (0.0)	0.101	0 (0.0)	3 (4.5)	1.0	3 (7.7)	0 (0.0)	0.242	3 (5.9)	0 (0.0)	0.548
Gender identity and dysphoria	16 (57.1)	21 (45.7)	0.472	15 (42.9)	22 (56.4)	0.352	4 (50)	33 (50)	1.0	19 (48.7)	18 (51.4)	1.0	29 (56.9)	8 (34.8)	0.131
History of psychiatric issues	6 (21.4)	4 (8.7)	0.164	6 (17.1)	4 (10.3)	0.502	1 (12.5)	9 (13.6)	1.0	6 (15.4)	4 (11.4)	0.740	8 (15.7)	2 (8.7)	0.715
Impact of gender identity on mental health	14 (50.0)	27 (58.7)	0.481	19 (54.3)	22 (56.4)	1.0	3 (37.5)	38 (57.6)	0.454	19 (48.7)	22 (62.9)	0.249	27 (52.9)	14 (60.9)	0.617
Suicidality	0 (0.0)	3 (6.5)	0.285	1 (2.9)	2 (5.1)	1.0	0 (0.0)	3 (4.5)	1.0	2 (5.1)	1 (2.9)	1.0	1 (2.0)	2 (8.7)	0.226
Social support	7 (25.0)	12 (26.1)	1.0	6 (17.1)	13 (33.3)	0.182	3 (37.5)	16 (24.2)	0.415	10 (25.6)	9 (25.7)	1.0	13 (25.5)	6 (26.1)	1.0
Coping Skills	2 (7.1)	3 (6.5)	1.0	4 (11.4)	1 (2.6)	0.183	0 (0.0)	5 (7.6)	1.0	4 (10.3)	1 (2.9)	0.361	4 (7.8)	1 (4.3)	1.0
Mental health history	4 (14.3)	4 (8.7)	0.467	7 (20.0)	1 (2.6)	0.023	2 (25.0)	6 (9.1)	0.206	5 (12.8)	3 (8.6)	0.714	6 (11.8)	2 (8.7)	1.0
History of hormone use	2 (7.1)	1 (2.2)	0.553	2 (5.7)	1 (2.6)	0.600	1 (12.5)	2 (3.0)	0.294	1 (2.6)	2 (5.7)	0.600	2 (3.9)	1 (4.3)	1.0
Substance use	1 (3.6)	2 (4.3)	1.0	2 (5.7)	1 (2.6)	0.600	0 (0.0)	3 (4.5)	1.0	2 (5.1)	1 (2.9)	1.0	3 (5.9)	0 (0.0)	0.548
Expectations of surgery	11 (39.3)	16 (34.8)	0.805	12 (34.3)	15 (38.5)	0.810	0 (0.0)	27 (40.9)	0.024	10 (25.6)	17 (48.6)	0.054	19 (37.3)	8 (34.8)	1.0
Motivations for surgery	7 (25.0)	17 (37.0)	0.318	11 (31.4)	13 (33.3)	1.0	2 (25.0)	22 (33.3)	1.0	15 (38.5)	9 (25.7)	0.321	14 (27.5)	10 (43.5)	0.190
Informed consent	9 (32.1)	22 (47.8)	0.229	11 (31.4)	20 (51.3)	0.102	5 (62.5)	26 (39.4)	0.267	15 (38.5)	16 (45.7)	0.638	20 (39.2)	11 (47.8)	0.612
Post-Operative Depression	22 (81.5)	44 (95.7)	0.093	30 (88.2)	36 (92.3)	0.698	7 (87.5)	59 (90.8)	0.573	33 (84.6)	33 (97.1)	0.113	43 (86.0)	23 (100)	0.090
Value Perception															
Yes	9 (32.1)	29 (63.0)	0.028	16 (45.7)	22 (56.4)	0.689	5 (62.5)	33 (50.0)	0.277	18 (46.2)	20 (57.1)	0.554	26 (51.0)	12 (52.2)	0.784
No	6 (21.4)	4 (8.7)		5 (14.3)	5 (12.8)		2 (25.0)	8 (12.1)		5 (12.8)	5 (14.3)		8 (15.7)	2 (8.7)	
Unsure	13 (46.4)	13 (28.3)		14 (40.0)	12 (30.8)		1 (12.5)	25 (37.9)		16 (41.0)	10 (28.6)		17 (33.3)	9 (39.1)	
Low Volume	36 (48.0)	39 (52.0)	0.014	50 (66.7)	25 (33.3)	< 0.001	13 (17.3)	62 (82.7)	1.0	41 (54.7)	34 (45.3)	0.632	54 (72.0)	21 (28.0)	0.597
High Volume	4 (18.2)	18 (81.8)		5 (22.7)	17 (77.3)		3 (13.6)	19 (86.4)		11 (50.0)	11 (50.0)		14 (63.6)	8 (36.4)	
Cisgender	33 (43.4)	43 (56.6)	0.629	45 (59.2)	31 (40.8)	0.472	15 (19.7)	61 (80.3)	0.111	42 (55.3)	34 (44.7)	1.0	53 (69.7)	23 (30.3)	0.801
TGNB	8 (36.4)	14 (63.6)		11 (50.0)	11 (50.0)		1 (4.5)	21 (95.5)		12 (54.5)	10 (45.5)		16 (72.7)	6 (27.3)	
Sessions Required (mean ± SD)	3.16 ± 2.61	3.07 ± 3.05	0.896	3.81 ± 3.26	2.50 ± 2.36	0.078	1.29 ± 0.81	3.33 ± 2.95	< 0.001	3.56 ± 3.42	2.60 ± 2.04	0.177	3.43 ± 3.03	2.45 ± 2.44	0.175
Assessments resulting in referral (mean ± SD)	88.95 ± 23.72	87.58 ± 18.25	0.808	83.40 ± 28.60	91.41 ± 8.86	0.129	90.83 ± 12.81	87.70 ± 20.70	0.719	82.94 ± 25.13	94.46 ± 6.38	0.027	86.45 ± 22.43	92.60 ± 8.82	0.308

Continuing education was also associated with requiring more sessions before writing a letter (mean (SD) 3.33 (2.95) vs. 1.29 (0.81); p=0.001). MHPs with clinical training had a higher proportion of assessments resulting in referral than those without clinical experience (94.46% (6.38%) vs. 82.94% (25.13%); p=0.027). A significantly higher proportion of MHPs who attended professional conferences as part of their training perceived value in the letter-writing process compared to those who had not (n=29/46 vs. n=9/28; p=0.028).

Table 3 presents assessment strategies, value perceptions, and outcomes stratified by provider gender and practice volume, with proportions calculated using respondents with data available for each item. TGNB MHPs were more likely than cisgender MHPs to view unrealistic expectations as a contraindication to referral (n=16/18 vs. n=29/56; p=0.005) and less likely to view substance use as a contraindication (n=6/18 vs. n=36/56; p=0.029). High-volume MHPs were also less likely to defer referral for substance use than low-volume MHPs, though this trend was not significant (n=8/21 vs. n=34/53; p=0.067).

**Table 3:** Assessment strategies and outcomes stratified by gender and volume subgroups Significant p-values (p <0.05) are bolded. Frequency data are reported as No. (%), with percentages calculated using respondents with available data for each item; therefore, denominators may vary row by row because missing responses were excluded from row-specific analyses. TGNB: Transgender and nonbinary

	Gender				P		Volume				P
	Cisgender 56		TGNB 18				Low 53		High 21		
Issues to address before referral	N	%	N	%			N	%	N	%	
Inability to give informed consent	43	76.8	16	88.9		0.333	43	81.1	16	76.2	0.750
Unrealistic expectations	29	51.8	16	88.9		0.005	32	60.4	13	61.9	1.0
Substance use issues	36	64.3	6	33.3		0.029	34	64.2	8	38.1	0.067
Compromised capacity	31	55.4	11	61.1		0.787	29	54.7	13	61.9	0.613
Inadequate knowledge	31	55.4	11	61.1		0.787	30	56.6	12	57.1	1.0
Value Perception	N	%	N	%			N	%	N	%	
Yes	32	57.1	6	33.3		0.208	24	45.3	14	66.7	0.174
No	6	10.7	4	22.2			7	13.2	3	14.3	
Unsure	18	32.1	8	44.4			22	41.5	4	19.0	
Permanent Barriers Exist	N	%	N	%			N	%	N	%	
Yes	24	42.9	5	27.8		0.165	21	39.6	8	38.1	0.330
No	17	30.4	10	55.6			17	32.1	10	47.6	
Unsure	15	26.8	3	16.7			15	28.3	3	14.3	
	Median	IQR	Median	IQR			Median	IQR	Median	IQR	
How many sessions before you write a letter?	2.0	1.5-4.5	1.5	1.0-2.0		0.003	2.8	1.5-5.0	1.5	1.0-2.0	< 0.001
	Mean	SD	Mean	SD			Mean	SD	Mean	SD	
Assessments resulting in referral	85.93	22.16	94.71	7.42		0.153	85.74	23.54	92.45	8.90	0.225

**Figure 4:**
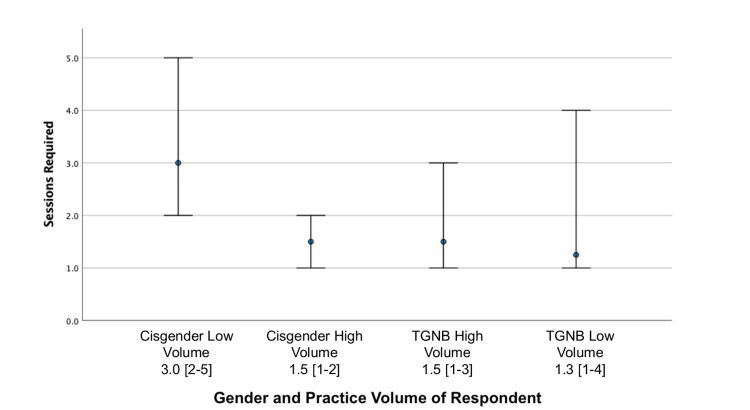
Required sessions prior to referral for gender-affirming surgery stratified by gender and volume of providers This figure demonstrates the required number of sessions required prior to respondents referring clients for gender-affirming surgery, as stratified by respondent gender and practice volume. Data is shown as median sessions required indicated by the blue midpoint. The hinges represent a 95% confidence interval.

Table 4 illustrates how the approach was influenced by provider gender identity and volume of evaluations performed. High-volume cisgender MHPs prioritized informed consent (n=9/12, 75%) and gender identity (n=9/12, 75%) more often than low-volume cisgender (n=14/63, 22.2%; n=19/63, 30.2%) and TGNB MHPs (n=3/12, 25%; n=4/12, 33.3%). High-volume TGNB MHPs were more likely to require unrealistic expectations to be addressed before referral than low-volume cisgender MHPs (n=8/10 vs. n=24/63; p=0.036). Low-volume cisgender MHPs required significantly more sessions before referral (mean (SD) 4.3 (3.4); median (IQR) 3 (2-6)) than high-volume cisgender (1.5 (0.4); 1.5 (1-2)), high-volume TGNB (1.9 (1.3); 1.5 (1-2)), and low-volume TGNB MHPs (1.8 (1.1); 1.3 (1-2.5); p=0.006). Overall, high-volume and TGNB MHPs required fewer sessions than low-volume and cisgender MHPs (Figure 4).

**Table 4:** Responses stratified by gender identity and evaluation volume of provider This table illustrates how the approach to evaluations was influenced by provider gender identity and volume of evaluations performed. Means that do not share subscripts significantly differ (p < 0.05) according to Bonferroni post-hoc tests. TGNB: Transgender and nonbinary.

	TGNB LV 12 (12.3)	Cisgender LV 63 (64.9)	TGNB HV 10 (10.3)	Cisgender HV 12 (12.3)	P
Important domains					
Trauma history	0	3 (4.7)	0	0	1.00
Gender identity and dysphoria	4 (33.3)_a_	19 (30.2)_a_	5 (50)_a,b_	9 (75)_b_	0.025
Psychiatric Issues or Diagnosis	2 (16.7)	7 (11.1)	0 (0)	1 (8.3)	0.698
Impact of gender identity on mental health	6 (50)	24 (38.1)	5 (50)	6 (50)	0.721
Suicidality	1 (8.1)	2 (3.2)	0	0	0.731
Social and Environmental Support	2 (16.7)	11 (17.5)	4 (40)	2 (16.7)	0.425
Coping Skills	1 (8.3)	4 (6.3)	0	0	1.0
Treatment history - Mental health	0	8 (12.7)	0	0	0.351
History of hormone use	1 (8.3)	2 (3.2)	0	0	0.731
Substance use, abuse, or dependence	1 (8.3)	1 (1.6)	1 (10)	0	0.219
Expectations of surgery	4 (33.3)	14 (22.2)	5 (50)	4 (33.3)	0.249
Motivations for surgery	2 (16.7)	16 (25.4)	2 (20)	4 (33.3)	0.847
Informed consent	3 (25)_a_	14 (21.9)_a_	5 (50)_a,b_	9 (75)_b_	0.002
Discuss Post-operative depression	7 (87.5)	39 (88.6)	9 (100)	11 (91.7)	0.856
Issues to address before referral					
Inability to give informed consent	8 (66.7)	35 (55.6)	8 (80)	8 (66.7)	0.466
Unrealistic expectations	8 (66.7)_a,b_	24 (37.5)_a_	8 (80)_b_	5 (41.7)_a,b_	0.037
Substance use issues					0.414
Compromised capacity	6 (50)	23 (35.9)	5 (50)	8 (66.7)	0.230
Inadequate knowledge	5 (41.7)	25 (39.7)	6 (60)	6 (50)	0.649
Value Perception					0.060
Yes	2 (22.2)	22 (50)	4 (44.4)	2 (22.2)	
No	1 (11.1)	6 (13.6)	3 (33.3)	1 (11.1)	
Unsure	6 (66.7)	16 (36.4)	2 (22.2)	6 (66.7)	
Permanent Barriers Exist					0.354
Yes	3 (33.3)	18 (40.9)	2 (22.2)	6 (50)	
No	5 (55.6)	12 (27.3)	5 (55.6)	5 (27.3)	
Unsure	1 (11.1)	14 (31.8)	2 (22.2)	1 (8.3)	
How many sessions before you write a letter? (mean ± SD)	1.81 ± 1.13_a_	4.27 ± 3.4_b_	1.94 ± 1.31_a_	1.45 ± 0.43_a_	0.006
Assessments resulting in referral (mean ± SD)	96.5 ± 5.05	83.8 ± 25.1	93.4 ± 8.9	91.8 ± 9.2	0.317

## Discussion

At our institution, which includes a multidisciplinary service line for transgender and gender-expansive health, community MHPs frequently seek guidance on conducting behavioral health assessments and writing readiness letters for GAS. Despite updated recommendations in WPATH SOCv8, insurance requirements have been slow to change and often still mandate MHP letters [7]. While SOCv8 broadened recommendations on who can provide referrals, its guidance on surgical readiness letter content remains vague, stating that “only one letter of assessment from a health care professional who has competencies in the assessment of transgender and gender diverse people is needed” and that “this letter needs to reflect the assessment and opinion from the team that involves both medical and mental health professionals” [7]. This ambiguity has led to continued requests for clearer structure and guidance in letter writing [8].

We sought to gather input from MHPs actively involved in the letter-writing process to better understand their backgrounds, experiences, and current approaches, and to gain insight from those most familiar with the process. Prior studies have documented patients’ negative experiences during GAS readiness assessments [12,17,22-26]. Our findings similarly revealed wide variability in evaluation and referral practices, underscoring the need for broader discussion about the purpose of readiness letters and the concept of gatekeeping in gender-affirming care.

We found that provider education was often initiated and reinforced through client interactions and community involvement. In the 2022 U.S. Transgender Survey, nearly one in four transgender adults reported having to educate their clinicians about transgender people [27]. Similarly, several respondents in our study cited learning from their patients as a key training experience. While American Psychiatric Association guidelines recommend that MHPs who lack certain competencies refer patients to more knowledgeable providers, the demand for GAS continues to exceed the supply of qualified providers [4,28-30]. As a result, patients are often placed in the position of educating providers rather than forming supportive therapeutic relationships [11,30].

The wide variation in educational backgrounds, therapeutic approaches, and the number of required visits between cisgender and TGNB MHPs underscores inconsistency within the letter-writing process. This variability can be interpreted in multiple ways. On one hand, tailoring therapy to individual needs can enable a patient-centered approach. As one respondent noted, “Each case is unique. If I am uncomfortable recommending surgery, I work with the person to address their complex mental health issues, which can often ebb and flow around a variety of identified concerns/stressors beyond surgery contemplation.”

However, fewer than half of providers reported receiving gender-specific education during their clinical training (Table 1), highlighting a gap in baseline competency for diagnosing and managing gender dysphoria. While no formal certification exists for MHPs to be able to write letters of surgical readiness, other subspecialty certifications (e.g., in eating disorders, addiction, or counseling) could serve as a model for standardized training [18,19]. Notably, findings of this study suggest that high-volume providers were more likely to have had continuing education and to require fewer sessions before referral, suggesting that structured training and experience enhance confidence and efficiency in assessment.

Proposing a standardized training pathway for MHPs inevitably raises concerns about gatekeeping in presurgical assessments. As one respondent noted, “My strong preference would be for an informed consent model, like other medical procedures. I feel tension between considerable discomfort with gate-keeping other people's transitions and wanting to help improve access to care by providing letters.” However, patient autonomy and structured presurgical readiness assessments can coexist. One way to ensure that assessments are both effective, beneficial, and patient-centered is to learn from the perspectives of experienced letter writers and MHPs who are TGNB themselves.

In our study, providers who engaged in formal learning through conferences or continuing education placed greater emphasis on establishing clear postoperative expectations. High-volume and TGNB MHPs prioritized assisting patients with perioperative navigation, addressing environmental stressors, and planning for financial and social support. MHPs with ongoing education or formal instruction were also more likely to see value in the assessment process, underscoring the importance of specific education in equipping MHPs with the skills and knowledge necessary to provide valuable presurgical assessments.

This study offers insight into current strategies used by MHPs conducting GAS readiness assessments. A notable limitation is the modest sample size and geographically restricted sampling, limiting generalizability. We were also unable to compare the experiences or practice volumes of non-respondents, introducing the possibility of non-response bias, although available demographic data did not differ significantly from those of respondents, except for fewer psychiatrists and psychiatric nurse practitioners. Although potential for selection and recall biases may exist due to sampling and survey design, a strength is that the instrument was developed by a diverse team actively engaged in gender-affirming care. Additionally, subgroup and nonresponse analyses helped mitigate potential biases from using a public directory. However, because subgroup analyses were conducted in a moderate sample, these findings should be interpreted as exploratory and hypothesis-generating rather than definitive. 

The survey also could not distinguish between evaluations for ongoing clients versus new patients seeking letters, which may influence reported session counts. Findings may not generalize to all MHP types; however, with SOCv8 expanding eligibility for letter writers to any master’s-level provider with adequate experience and training, these results are particularly relevant for providers who are new to GAS evaluations. Future studies should include larger samples, representation from more states, and updated surveys aligned with the most recent WPATH SOC [7].

## Conclusions

This study highlights substantial variability in presurgical evaluations of TGNB patients seeking GAS within a large metropolitan population, driven by differences in evaluator training, experiences, and approach. This variability includes inconsistent session requirements and referral contraindications, reflecting a lack of standardization in the assessment process. Although most evaluations resulted in referral, this inconsistency underscores the need for clearer, more structured guidelines. The higher evaluation volume among TGNB-identifying MHPs and those engaged in continuing education or mentorship further emphasizes the importance of training and experience in the letter-writing process. These findings can help inform future guidelines for surgical readiness assessments to promote consistent, equitable, and patient-centered care prior to gender-affirming surgery.

## Appendices

Full survey

Psychosocial Domains Assessed During Gender Affirming Surgery Referrals

We are asking you to take part in a research study being done by [removed]. If you choose to be in the study, you will complete a survey. This survey will help us learn more about how mental health professionals (MHPs) determine someone's candidacy for gender-affirming surgery (GAS) referral. The survey will take about 15 minutes to complete. You can skip questions that you do not want to answer or stop the survey at any time. The survey is anonymous, and no one will be able to link your answers back to you.

Benefits: Being in this research study will not benefit you directly. Participants are eligible to enter a raffle for one $200 dollar Amazon gift card. We hope that your participation in the study will provide helpful information for your mental health professional colleagues when they are caring for patients interested in GAS. This can benefit the transgender community as they work to access GAS services. Risks: The primary risk presented by this research study is breaches of privacy, but no demographic information will be collected, so responses cannot be linked to specific individuals.

Questions about the study? Please contact [removed]: If you have questions or concerns about your rights as a research participant, you can call [removed]. This study has been approved by the [removed] Institutional Review Board. If you want to 8. participate in this study, please continue below. Thank you!

1. What terms best express how you describe your gender identity?

O Woman

O Man

O Non-binary

O Transgender man/Female-to-male (FTM)

O Transgender woman/Male-to-female (MTF)

O Gender non-binary/Genderqueer/Gender nonconforming

O Agender

O None of these describes me

O Prefer not to answer

2, Race and Ethnicity (select all that apply):

O Native Hawaiian or Other Pacific Islander

O American Indian or Alaska Native

O Asian

O Black or African American

O White

O Hispanic or Latino/Latina/Latinx

3. Age, in years: _____

4. Professional Category:

O Psychiatrist

O Psychologist

O Marriage/family therapist

O Counselor

O Social worker

O Psychiatric nurse practitioner

O Family Medicine

5. What is your highest level of education?

O PhD/PsyD/EdD

O MSW/LCSW

O MA/MS

 O MD/DO

O RN/MSN/CRNP

O LCMFT

O Other/Not Listed

6. What, if any, training have you had in caring for transgender patients?

O Professional conference (Continuing education, Mentorship)

O Training during residency, fellowship, internship or other clinical setting

O Graduate Course

O Other

O Not applicable

7. Please Specify: _____

(a) Are you familiar with the World Professional Association for Transgender Health (WPATH) Standards of Care?

O Yes

O No

(b) Do you practice under the WPATH Standards of Care?

O Yes

O No

O I use another model

8. Please Specify: _____

(a) How would you describe your practice?                                    

O Public

O Private

(b) Is your practice primarily:

O Urban

O Suburban

O Rural

O Telehealth

9. Years in practice: _____

10. Years conducting evaluations: _____

11. On average, how many evaluations for gender affirming surgery do you conduct per year?

12. Assessment procedures used in preoperative psychological evaluation of candidates for gender affirming surgery:

Clinical interview: Never, Almost Never, Sometimes, Often, Almost Always

Symptom inventories/screening tools: Never, Almost Never, Sometimes, Often, Almost Always

Tests of cognitive function: Never, Almost Never, Sometimes, Often, Almost Always

Objective personality/psychopathology tests: Never, Almost Never, Sometimes, Often, Almost Always

Projective personality tests: Never, Almost Never, Sometimes, Often, Almost Always

13. Domains assessed

Background History: Never, Almost Never, Sometimes, Often, Almost Always

Gender identity and dysphoria: Never, Almost Never, Sometimes, Often, Almost Always

Psychiatric issues or diagnoses: Never, Almost Never, Sometimes, Often, Almost Always

Treatment history - mental health: Never, Almost Never, Sometimes, Often, Almost Always

Impact of gender identity on mental health: Never, Almost Never, Sometimes, Often, Almost Always

Suicidality or passive death wish: Never, Almost Never, Sometimes, Often, Almost Always

Social/Environmental support: Never, Almost Never, Sometimes, Often, Almost Always

Coping skills: Never, Almost Never, Sometimes, Often, Almost Always

History of hormone use: Never, Almost Never, Sometimes, Often, Almost Always

Substance use, abuse, or dependence: Never, Almost Never, Sometimes, Often, Almost Always

Expectations of surgery: Never, Almost Never, Sometimes, Often, Almost Always

Motivations and Rationale for surgery: Never, Almost Never, Sometimes, Often, Almost Always

Informed Consent (Capacity and Knowledge): Never, Almost Never, Sometimes, Often, Almost Always

Prior experience with surgery: Never, Almost Never, Sometimes, Often, Almost Always

Other: Never, Almost Never, Sometimes, Often, Almost Always

14. Please specify: ____

(a) What are the most important things you assess during pre-op eval? Pick up to 3:

O Background history

O History of trauma/abuse

O Medical history

O Gender identity and dysphoria

O Psychiatric issues or diagnoses

O Treatment history - Mental health

O Impact of gender identity on mental health

O Suicidality (ideation/history of attempts) or Passive Death Wish

O Social/environmental support

O Coping Skills

O History of hormone replacement therapy

O Substance Use/Abuse/Dependence

O Expectations of surgery

O Motivations/rationale for surgery

O Informed Consent (Capacity & Knowledge)

O Patient's prior experience with surgery

O other

(b) Is post-op depression discussed?                                             

O Yes

O No

15. How many hours do you spend with the patient conducting the evaluation?

16. How many sessions before you write a letter?

17. Do you perceive any value in the current referral process?

O Yes

O No

O Unsure

18. Which of the following issues (if any) require treatment/ resolution before referral for surgery?

O Substance use/abuse/dependence

O Psychotic disorders

O Depression

O Suicide (ideation, history of attempts)

O Passive Death Wish D Personality disorders D Anxiety disorders

O Psychiatric hospitalizations

O Bipolar disorder

O Other mental health issues

O Inability to give informed consent

O Compromised capacity

O Inadequate knowledge

O Other informed consent issues

O Environmental/Social Barriers to Success

O Support system

O Current stressors

O Other environmental/social barriers

O Internal Barriers to Success

O Maladaptive coping

O impulsivity

O Other internal barriers

O Adherence Problems

O Demonstrated non-adherence

O Potential non-adherence

O Other adherence problems

O Problematic Expectations/Rationale for Surgery

O Unrealistic expectations

O Non-health rationale for surgery

O Other expectations/rationale issues

O Other

19. Please specify: ____

(a) Are there any issues that are permanent barriers to referral for gender affirming surgery?

O Yes

O No

O Unsure

(b) What are they?

20. Do you make explicit recommendations in your referral letter?

O Yes

O No

21. Please estimate the percentage of people you recommend:

Unconditional referral for surgery

Deferring until specific issues are addressed

Foregoing surgery altogether

22. Please share any other comments or concerns: ____
